# Factors influencing glycaemic stability after neonatal hypoglycaemia and relationship to neurodevelopmental outcome

**DOI:** 10.1038/s41598-019-44609-1

**Published:** 2019-05-31

**Authors:** Nataliia Burakevych, Christopher J. D. McKinlay, Deborah L. Harris, Jane M. Alsweiler, Jane E. Harding

**Affiliations:** 1 Liggins Institute, University of Auckland, Private Bag 92019, Auckland, 1010 New Zealand; 2 Department of Paediatrics: Child and Youth Health, University of Auckland, Auckland, New Zealand; 3 Newborn Intensive Care Unit Waikato District Health Board P.O. Box 934, Hamilton, New Zealand

**Keywords:** Paediatric research, Risk factors

## Abstract

Higher and unstable glucose concentrations in the first 48 hours in neonates at risk of hypoglycaemia have been associated with neurosensory impairment. It is unclear what defines and contributes to instability. This was a prospective study of term and late preterm babies (N = 139) born at risk of neonatal hypoglycaemia who had interstitial glucose (IG) monitoring and ≥1 hypoglycaemic episode <48 hours after birth (blood glucose concentration <2.6 mmol/l [<47 mg/dl]). For 6-hour epochs after each hypoglycaemic episode, masked IG parameters (time to reach maximum IG concentration [hours]; range, average, maximum and minimum IG concentrations; proportion of IG measurements outside the central band of 3–4 mmol/l [54–72 md/dl]; and total duration [hours] of IG concentrations <2.6 mmol/l) were analysed in tertiles and related to: (i) glycaemic instability in the first 48 hours (defined as the proportion of blood glucose concentrations outside the central band in the first 48 hours); (ii) risk factors and treatment for each episode; and (iii) risk of neurosensory impairment at 4.5 years, or at 2 years if a child was not seen at 4.5 years. Glycaemic instability in the first 48 hours was related to IG instability after hypoglycaemia. Risk factors for hypoglycaemia were not related to IG parameters. Treatment with intravenous dextrose was associated with higher IG maximum and range, and lower minimum compared to treatment with dextrose gel plus breast milk, breast milk alone or formula alone. The risk of neurosensory impairment was increased with both shorter and longer time to reach maximum epoch IG (P = 0.04; lower tertile [0.4–2.2 hours] vs middle [2.3–4.2 hours] OR 3.10 [95% CI 1.03; 9.38]; higher tertile [4.3–6.0 hours] vs middle OR 3.07; [95% CI 1.01; 9.24]). Glycaemic response to hypoglycaemia contributes to overall glycaemic instability in newborns and is influenced by treatment. Slow or rapid recovery of hypoglycaemia appears to be associated with neurosensory impairment.

## Introduction

Neonatal hypoglycaemia is a common condition described as a failure of metabolic adaptation to the postnatal environment^1–3^. At birth the continuous supply of glucose is interrupted and successful transition to neonatal life requires adequate fuel stores, mature glycogenolytic and gluconeogenic pathways and hormonal homeostatic systems^4,5^. Babies born to diabetic mothers, or who are born preterm, small or large often have impaired metabolic adaptation and are at risk of neonatal hypoglycaemia.

Severe or symptomatic neonatal hypoglycaemia is a known cause of brain injury, but thresholds for diagnosis and treatment of asymptomatic neonatal hypoglycaemia are controversial^6^. We have previously shown in a large prospective cohort that neonatal hypoglycaemia, when treated to maintain blood glucose at or above 2.6 mmol/l (47 mg/dl), may affect specific neurological functions including executive function and visual-motor coordination^7^. Further, babies with higher or less stable blood glucose concentrations in the first 48 hours had higher risk of neurosensory impairment^8^. These associations were strongest in babies who had experienced hypoglycaemia and were treated with dextrose, raising concern that glycaemic responses to treatment may influence long-term neurodevelopmental outcome after hypoglycaemia. However, it was not clear if the instability and adverse outcome were related to different responses of babies who were treated similarly, or to different treatments, and which of the parameters that define instability were related to the outcome.

Therefore, we undertook a detailed analysis of interstitial glucose (IG) concentrations following episodes of neonatal hypoglycaemia. Data were collected using continuous glucose monitoring, which is not used in routine care, but provides blinded readings of IG concentrations every five minutes^9,10^ allowing in-depth investigation of glycaemic responses. The aim of this study was to investigate [1] the association between stability of blood glucose in the first 48 hours and interstitial glucose parameters following hypoglycaemia; [2] the effects of neonatal risk factors, gestational age at birth, feeding and dextrose treatment on changes in glucose parameters following hypoglycaemia; [3] the association between interstitial glucose parameters following hypoglycaemia and neurosensory impairment at 2 or 4.5 years.

## Materials and Methods

### Study design

This was a prospective cohort study of babies born at risk of neonatal hypoglycaemia at Waikato Women’s Hospital, Hamilton, New Zealand between 2006 and 2010. Babies were recruited to two neonatal studies, BABIES^11^ and Sugar Babies^12^ if they had one or more risk factor for neonatal hypoglycaemia.: born to diabetic mother, preterm (32–36 completed weeks’ gestation), small (<2500 g or birthweight <10^th^ centile), large (>4500 g or >90^th^ centile) or other conditions (e.g., poor feeding, respiratory distress). Hypoglycaemia was defined as blood glucose concentration <2.6 mmol/l (<47 mg/dl).

### Neonatal management

Breast feeding was encouraged and infant formula or expressed breast milk was given until adequate breast milk was available according to maternal preference. All babies born <35 weeks’ gestation were routinely admitted to the Neonatal Intensive Care Unit (NICU).

Babies who became hypoglycaemic were treated with feeding (breast milk or formula according to mother’s preference), 40% dextrose gel massaged into the buccal mucosa followed by feeding or intravenous 10% dextrose (2 ml/kg bolus over 10 minutes and infusion 60–90 ml/kg/day [4–6 mg/kg/min]). Formula feeds were given at 7.5 ml/kg on day one (60 ml/kg per day) and 11 ml/kg on day two (90 ml/kg per day).

### Measures

Blood glucose concentration was measured by heel-prick at one hour of age, then every two to four hours before feeding for 24 hours, and every 6 to 8 hours for the next 24 hours, or until stable. In babies who received gel, blood glucose concentration was also measured 30 minutes after treatment. Babies who received intravenous dextrose had blood glucose concentration measured every 4 hours for 12 hours, and then as clinically indicated. All blood glucose measurements were analysed using glucose oxidase (Radiometer, ABL800 FLEX, Copenhagen, Denmark). A continuous interstitial glucose monitor (CGMS® system gold ™ Medtronic, MiniMed, Northridge, CA, USA) was inserted in the lateral thigh soon after birth^11^, and monitoring continued for at least 48 hours. Data from the continuous glucose monitors were downloaded at the end of the monitoring period and were not available to clinical staff, so did not affect clinical management. These data were recalibrated to true blood glucose concentrations, as previously described^13^.

### Assessment at 2 and 4.5 years

Children underwent comprehensive neuropsychometric testing of cognitive ability, language, executive function, visual and motor function, and social emotional status at 2 and 4.5 years’ corrected age, as previously described^7,8^. Parents completed questionnaires on socio-demographic characteristics of their households.

Neurosensory impairment at 2 years^8,14^ was defined as one or more of: developmental delay (Bayley Scales of Infant and Toddler Development, third edition^15^ cognitive or language composite score <85), motor difficulty (Bayley motor composite score <85 or cerebral palsy), visual impairment or hearing impairment requiring hearing aids, executive function score or motion coherence threshold worse than 1.5 SD from the cohort mean^16^.

Neurosensory impairment at 4.5 years was defined as one or more of: cognitive delay (Wechsler Preschool and Primary Scale of Intelligence, 3^rd^ edition full scale IQ < 85), motor difficulty (Movement Assessment Battery for Children, second edition total score <15^th^ centile or cerebral palsy), visual impairment (best visual acuity >0.5 logMAR), visual-motor difficulty (Beery-Buktenica Developmental Test of Visual-Motor Integration, sixth edition VMI score <85), hearing impairment (requiring hearing aids), executive function score or motion coherence threshold worse than 1.5 SD from the cohort mean.

### Statistical analysis

Analysis was performed using SAS version 9.4 (SAS Institute). An episode of hypoglycaemia was defined as one or more consecutive blood glucose concentrations <2.6 mmol/l (<47 mg/dl). The analysis reported here was restricted to babies who experienced at least one episode of hypoglycaemia in the first 48 h after birth and who had CGM *in situ* from the onset of the episode.

We analysed IG concentrations for 6 hours epochs after the onset of a hypoglycaemic episode. For each epoch, the following parameters were defined: number of blood glucose measurements <2.6 mmol/l (<47 mg/dl); time to reach maximum IG concentration (hours); range, average, maximum and minimum IG concentrations; proportion of IG measurements outside the 3 to 4 mmol/l (54 to 72 md/dl) central band^8^; and total duration (hours) of IG concentrations <2.6 mmo/l (<47 mg/dl).

The primary risk factor for neonatal hypoglycaemia was defined using the following hierarchical order: infants of diabetic mothers, preterm, small, large and other. Epochs were classified according to dextrose treatment received during the 6-hour period, prioritised as intravenous dextrose, buccal dextrose gel or no dextrose, and by feed received, prioritised as formula and breast milk (expressed or breastfeed).

To assess the association between stability of blood glucose concentrations in the first 48 hours and interstitial glucose parameters following hypoglycaemia (aim 1), babies were divided into tertiles of proportion of blood glucose concentrations in the first 48 hours outside the central range of 3–4 mmol/l (54–72 mg/dl). Epoch IG parameters were compared among tertiles using generalised mixed linear models, accounting for multiple epochs per baby (random effect).

To assess the effect of neonatal risk factors, gestational age at birth, feeding and dextrose treatment on IG concentrations following hypoglycaemia (aim 2), epoch IG parameters were compared among different risk, gestational age and treatment groups using generalised mixed linear models, accounting for multiple epochs per baby (random effect). Additional multivariate analysis was performed for epochs with IV dextrose treatment to assess associations between IG parameters and IV bolus, glucose delivery rate (GDR, mg/kg/min) and buccal gel, with adjustment for time of onset of IV dextrose.

To assess the association between IG concentrations following hypoglycaemia and neurosensory impairment (aim 3), children were divided into tertiles for each epoch IG parameter. For those with multiple epochs, the first epoch was used for quintile classification^17^. Rates of neurosensory impairment were compared among tertiles using generalised linear models, adjusted for socio-economic status, gestational age and birth weight Z score. Because our aim was to investigate the potential impact of glycaemic patterns after hypoglycaemia on neurodevelopment independent of hypoglycaemia severity, we also adjusted for blood glucose concentration at the onset of the epoch. For this analysis, epoch IG parameters can be interpreted as measures of rate of change or stability of glucose concentrations after hypoglycaemia. Neurosensory status was based on outcomes at 4.5 years, or at 2 years if a child was not assessed at 4.5 years. Exposure effects are presented as odds ratios with 95% confidence intervals (CI).

Alpha was set at 0.05 for all analyses. Dunnett or Tukey adjustment was performed for *post hoc* pairwise comparisons, as appropriate (different letters indicate significant pairwise difference). Epoch IG duration <2.6 mmol/l was logarithmically transformed for analysis.

### Ethics

The Study was approved by the Northern Y Ethics Committee. Caregivers provided written informed consent for the neonatal and follow-up studies. All assessments were performed in accordance with relevant guidelines and regulations.

## Results

A total of 614 babies were recruited to the two neonatal studies (two babies were in both). Cohort and neonatal outcomes have been reported previously^11,12,18,19^. A total of 404 children were assessed at two years’ corrected age (77% of those eligible) and 477 (79% of eligible) at 4.5 years. There were 201 6-hour hypoglycaemic epochs with complete IG monitoring available for analysis from 139 babies, of whom 66 (47%) were boys, 11 (8%) were born at 32–34 weeks’ gestational age, 49 (35%) at 35–36 weeks’ and 79 (57%) at term (Table 1). One-quarter of babies (35) had multiple epochs but these babies were not significantly different from those with a single epoch in ethnicity (P = 0.06), socioeconomic status (P = 0.17), gestational age at birth (P = 0.10), sex (P = 0.74) or primary risk factors for hypoglycaemia (P = 0.74). Of the 139 babies included in this study, 112 (81%) completed neurodevelopmental assessment at 4.5 years of age and 27 (19%) were assessed only at 2 years.

**Table 1:** Cohort characteristics.

Characteristic	Cohort	Gestational age groups (weeks)		
		32–34	35–36	≥37
Number of 6-hour epochs	201	14	71	116
Number of babies	139	11	49	79
Primary risk factor:***				
IDM	46 (33)	4 (36)	7 (14)	35 (44)
Preterm	48 (35)	7 (64)	41 (84)	0 (0)
Small	28 (20)	0 (0)	1 (2)	27 (34)
Large	8 (6)	0 (0)	0 (0)	8 (10)
Other	9 (6)	0 (0)	0 (0)	9 (11)
Boys	66 (47)	5 (45)	26 (53)	35 (44)
Ethnicity:				
New Zealand European	76 (55)	8 (73)	23 (47)	45 (57)
Māori	54 (39)	2 (18)	24 (49)	28 (35)
Pacific Islander	3 (2)	0 (0)	1 (2)	2 (3)
Asian	6 (4)	1 (9)	1 (2)	4 (5)
Admitted to Neonatal Intensive Care Unit**	82 (59)	11 (100)	31 (63)	40 (51)
Feeding/treatment:***				
Breast milk only	25 (18)	0 (0)	10 (20)	15 (19)
Formula only	24 (17)	1 (9)	11 (22)	12 (15)
Dextrose gel + breast milk	32 (23)	0 (0)	8 (16)	24 (30)
Dextrose gel + formula	30 (22)	1 (9)	14 (29)	15 (19)
IV dextrose	28 (20)	9 (82)	6 (12)	13 (16)
Neurosensory impairment	49 (41)	3 (33)	19 (49)	27 (38)

Data are number (percent). IDM, infant of a diabetic mother, IV, intravenous. **P < 0.01, ***P < 0.001 for comparison among gestational age groups (Fisher’s exact test). Neurosensory status based on assessment at 4.5 years, or at 2 years if child not seen at 4.5 years (for description of neurosensory impairment see methods).

### Blood glucose stability

Babies with more unstable blood glucose concentrations (outside central range of 3–4 mmol/l) in the first 48 hours had lower blood glucose concentrations at the onset of the epoch (tertile three vs one mean difference [MD] −0.2 mmol/l [95% CI −0.4; −0.08], P = 0.002; and tertile two vs one MD −0.2 mmol/l; [95% CI −0.3; −0.02], P = 0.02). They also had more variable interstitial glucose parameters after hypoglycaemia, with lower epoch IG minimum (tertile three vs one mean difference [MD] −0.2 mmol/l [95% CI −0.4; −0.03], P = 0.02; and tertile two vs one MD −0.2 mmol/l; [95% CI −0.4; −0.05], P = 0.01), and higher epoch IG maximum (tertile three vs two: MD 0.5 mmol/l [95% CI 0.04; 1.1], P = 0.03) and range (tertile three vs one MD 0.7 mmol/l [95% CI 0.1; 1.2], P = 0.01, and tertile three vs two MD 0.6 mmol/l [0.02; 1.1], P = 0.04) (Table 2). They also had a higher proportion of epoch IG concentrations outside the central band of 3 to 4 mmol/l (tertile 3 vs 1 MD 0.29 [95% CI 0.17; 0.41], P < 0.0001; tertile two vs one MD 0.18 [95% CI 0.06; 0.30], P = 0.03) and more time with epoch IG below 2.6 mmol/l (tertile 3 vs 1 MD 0.57 hours [95% CI 0.17; 0.97], P = 0.003; tertile two vs one MD 0.62 hours [95% CI 0.22; 1.02], P = 0.002) (Table 2).

**Table 2:** Interstitial glucose parameters in 6-hour epochs following hypoglycemia and blood glucose stability in the first 48 hours after birth.

	Proportion of blood glucose concentrations outside central band (3–4 mmol/l) in first 48 hours		
	Tertile One (0.18–0.50)	Tertile Two (0.51–0.64)	Tertile Three (0.65–1.00)
Number of 6 h-epochs (babies)	57 (49)	53 (41)	58 (41)
Blood glucose concentration at the onset of the epoch**	2.3 (0.2)^a^ 2.4 [2,2; 2.5]	2.2 (0.3)^b^ 2.2 [2.0; 2.4]	2.1 (0.5)^b^ 2.3 [1.9; 2.4]
Number of blood glucose concentrations <2.6 mmol/l within epoch	1 [1;1]	1 [1;2]	1 [1;2]
Hours to maximum IG	3.4 (1.7) 3.3 [1.9;5.2]	3.6 (1.7) 3.7 [2.4;5.3]	3.2 (1.6) 2.7 [2.1;4.3]
Average IG	3.2 (0.3) 3.2 [3.0;3.4]	3.0 (0.5) 3.0 [2.7;3.2]	3.3 (0.8) 3.1 [2.7;3.6]
Maximum IG*	3.8 (0.5)^a^ 3.8 [3.4; 4.0]	3.7 (1.0)^a^ 3.5 [3.3;3.8]	4.2 (1.3)^b^ 3.8 [3.1;4.8]
Minimum IG**	2.3 (0.3)^a^ 2.4 [2.2; 2.5]	2.1 (0.3)^b^ 2.2 [1.9;2.3]	2.1 (0.5)^b^ 2.2 [1.9;2.4]
IG range**	1.5 (0.6)^a^ 1.4 [1.2;1.7]	1.6 (1.1)^a^ 1.3 [1.1;1.9]	2.1 (1.3)^b^ 1.7 [1.1;2.7]
Proportion IG outside central band of 3–4 mmol/l***	0.42 (0.25)^a^ 0.36 [0.19;0.61]	0.60 (0.28)^b^ 0.60 [0.36; 0.82]	0.69 (0.25)^b^ 0.73 [0.51;0.94]
Duration (hours) IG <2.6 mmol/l***	0.82 (0.74)^a^ 0.55 [0.32;0.99]	1.51 (1.23)^b^ 1.32 [0.57;1.86]	1.60 (1.43)^b^ 1.18 [0.49;2.32]

Data are number, median [interquartile range] or mean (standard deviation), unless otherwise stated. Glucose concentrations expressed as mmol/l. IG, interstitial glucose. *P < 0.05, **P < 0.01, ***P < 0.001 for comparison among tertiles; letters indicate pairwise comparisons (Dunnett adjustment, tertile one as referent).

### Neonatal risk factors and gestational age

Epoch IG parameters were similar among neonatal risk groups (Table 3). Babies born at 32–34 weeks’ gestational age had lower blood glucose concentrations at epoch onset than babies born at term (MD −0.34 mmol/l [95% CI −0.55; −0.13], P = 0.0006) (Table 4). They also had significantly higher epoch IG average (MD 0.59 mmol/l [95% CI 0.20; 1.00], P = 0.002), maximum (MD 0.86 mmol/l [95% CI 0.12; 1.61], P = 0.02) and range (MD 1.02 mmol/l [95% CI 0.24; 1.80], P = 0.007) than term babies. IG parameters were similar in babies born at 35–36 and ≥37 weeks’ gestational age (Table 4).

**Table 3:** Primary neonatal risk groups and interstitial glucose parameters following hypoglycemia.

Number of 6-hour epochs (babies)	Total	IDM	Preterm	Small	Large	Other
	201 (139)	56 (46)	73 (48)	49 (28)	11 (8)	12 (9)
Blood glucose concentration at the onset of the epoch	2.2 (0.3) 2.3 [2.1;2.5]	2.1 (0.4) 2.2 [2.1; 0.4]	2.2 (0.3) 2.3 [2.1;2.4]	2.2 (0.3) 2.4 [2.1;2.5]	2.2 (0.3) 2.3 [2.0;2.5]	2.2 (0.2) 2.2 [2.1;2.5]
Number of blood glucose concentrations <2.6 mmol/l per epoch	1 [1;2]	1 [1;2]	1 [1;2]	1 [1;2]	1 [1;2]	1 [1;2]
Hours to maximum IG	3.3 (1.7) 3.0 [2.0; 4.9]	3.4 (1.7) 3.0 [2.2;5.3]	3.5 (1.8) 3.2 [2.0;5.3]	3.0 (1.5) 2.7 [1.6;4.4]	3.6 (1.3) 3.7 [2.7;4.2]	3.5 (1.7) 3.9 [1.6;4.8]
Average IG	3.2 (0.6) 3.1 [2.8;3.5]	3.2 (0.7) 3.1 [2.8;3.5]	3.3 (0.6) 3.2 [2.9;3.6]	3.3 (0.7) 3.1 [2.9;3.6]	3.1 (0.4) 3.0 [2.9;3.5]	3.1 (0.4) 3.2 [2.7;3.3]
Maximum IG	4.1 (1.1) 3.7 [3.4;4.5]	3.9 (1.0) 3.6 [3.3;4.4]	4.2 (1.2) 3.7 [3.5;4.5]	4.3 (1.3) 3.9 [3.4;4.8]	3.9 (0.5) 3.8 (3.4;4.4)	3.7 (0.5) 3.8 [3.3;3.9]
Minimum IG	2.2 (0.4) 2.2 [2.0;2.4]	2.1 (0.4) 2.2 [1.9;2.4]	2.2 (0.3) 2.2 [2.1;2.4]	2.2 (0.4) 2.3 [2.0;2.4]	2.2 (0.3) 2.2 [2.0;2.3]	2.2 (0.2) 2.2 [2.0;2.4]
IG range	1.9 (1.2) 1.6 [1.2;2.2]	1.8 (1.0) 1.6 [1.1;2.3]	1.9 (1.2) 1.6 [1.2;2.2]	2.1 (1.5) 1.6 [1.2;2.7]	1.7 (0.5) 1.7 [1.4;1.9]	1.5 (0.5) 1.4 [1.2;1.6]
Proportion IG outside central band of 3–4 mmol/l	0.57 (0.28) 0.61 [0.35;0.77]	0.59 (0.27) 0.62 [0.40;0.79]	0.55 (0.30) 0.58 [0.27;0.82]	0.60 (0.27) 0.66 [0.39;0.79]	0.55 (0.24) 0.57 [0.38;0.75]	0.51 (0.33) 0.47 [0.23;0.79]
Duration (hours) IG <2.6 mmol/l	1.32 (1.23) 0.88 [0.41;1.76]	1.42 (1.41) 0.88 [0.40;1.85]	1.12 (0.96) 0.81 [0.41;1.42]	1.45 (1.42) 0.88 [0.40;2.16]	1.49 (1.02)1.28 [0.88;2.05]	1.43 (1.08) 1.16 [0.79;2.08]

Data are number, median [interquartile range] or mean (standard deviation). Glucose concentrations expressed as mmol/l. IG, interstitial glucose concentration. IDM, infant of a diabetic mother. No significant differences in glucose parameters among neonatal risk groups.

**Table 4:** Gestational age groups and interstitial glucose parameters following hypoglycemia.

	Total	Gestational age groups (weeks)		
		32–34	35–36	≥37
Number of 6-hour epochs (babies)	201 (139)	14 (11)	71 (49)	116 (70)
Blood glucose at the onset of the epoch**	2.2 (0.3) 2.3 [2.1; 2.5]	1.9 (0.7)^b^ 2.1 [1.7; 2.2]	2.3 (0.2)^a^ 2.3 [2.1; 2.4]	2.2 (0.3)^a^ 2.3 [2.1; 2.5]
Number of blood glucose concentrations <2.6 mmol/l per epoch	1 [1; 2]	1 [1; 2]	1 [1; 2]	1 [1; 2]
Hours to maximum IG*	3.3 (1.7) 3.0 [2.0; 4.9]	2.4 (1.1) 2.2 [1.7; 2.5]	3.7 (1.8) 3.5 [2.2; 5.6]	3.2 (1.6) 3.0 [2.0; 4.6]
Average IG**	3.2 (0.6) 3.1 [2.8; 3.5]	3.7 (0.9)^b^ 3.7 [3.0; 4.4]	3.3 (0.6)^a^ 3.2 [2.9; 3.6]	3.1 (0.6)^a^ 3.1 [2.8; 3.4]
Maximum IG*	4.1 (1.1) 3.7 [3.4;4.5]	5.0 (1.6)^b^ 4.8 [3.7; 5.6]	4.1 (1.1)^a^ 3.7 [3.4; 4.5]	4.0 (1.0)^a^ 3.7 [3.4; 4.2]
Minimum IG*	2.2 (0.4) 2.2 [2.0;2.4]	2.0 (0.7) 2.2 [1.7; 2.5]	2.2 (0.3) 2.3 [2.1; 2.4]	2.2 (0.3) 2.2 [2.0; 2.4]
IG range*	1.9 (1.2) 1.6 [1.2;2.2]	3.1 (1.7)^b^ 2.5 [2.0; 4.3]	1.9 (1.1)^a^ 1.6 [1.2; 2.2]	1.8 (1.1)^a^ 1.6 [1.1; 2.1]
Proportion IG outside central band of 3-4 mmol/l	0.57 (0.28) 0.61 [0.35;0.77]	0.66 (0.27) 0.73 [0.46; 0.85]	0.55 (0.29) 0.58 [0.27; 0.82]	0.58 (0.28) 0.61 [0.37; 0.76]
Duration (hours) IG <2.6 mmol/l	1.32 (1.23) 0.88 [0.41;1.76]	1.06 (1.47) 0.52 [0.32; 1.34]	1.13 (0.96) 0.87 [0.41; 1.52]	1.47 (1.33) 1.04 [0.41; 2.04]

Data are number, median [interquartile range] or mean (standard deviation). Glucose concentrations are expressed as mmol/l. IG, interstitial glucose concentration. *P < 0.05, **P < 0.01 for comparisons among gestational age groups; letters indicate pairwise comparisons (Dunnett adjustment, ≥37 weeks as referent).

### IG response to feeding and treatment

Intravenous dextrose was given to 9/11 (82%) babies born at 32–34 weeks’, 6/49 (12%) at 35–36 weeks’ and 13/79 (16%) at term (Table 1). Analysis of the effect of feeding and treatment was therefore restricted to babies born at ≥35 weeks’ gestational age (187 epochs in 128 babies).

Epochs were compared among five hierarchical feeding and treatment groups: breast milk only, formula, breast milk plus buccal dextrose gel, formula plus buccal dextrose gel, and IV dextrose. Breast milk alone was provided in 30/187 (16%) epochs, formula alone in 39/187 (21%) and breast milk plus gel in 44/187 (24%) epochs (Table 5). Sex, ethnicity, socio-economic status, primary neonatal risk factor and size at birth did not differ among these groups (data not shown). However, babies treated with formula plus dextrose gel and those treated with IV dextrose were more likely than other treatment groups to be admitted NICU (76% and 100% of babies, respectively, P < 0.001).

**Table 5:** Effect of feeding and treatment on interstitial glucose parameters in 6-hour epochs following hypoglycemia.

	Breast milk	Formula	Dextrose gel and breast milk	Dextrose gel and formula	IV dextrose
Number of 6-hours epochs (babies)^†^	30 (25)	39 (23)	44 (32)	38 (29)	36 (19)
Blood glucose at the onset of the epoch	2.3 (0.2)^a^ 2.4 [2.3; 2.5]	2.3 (0.3)^a,b^ 2.4 [2.1; 2.5]	2.3 (0.3)^a,b^ 2.3 [2.1; 2.5]	2.2 (0.3)^a,b^ 2.3 [2.1; 2.5]	2.1 (0.4)^b^ 2.2 [2.0; 2.4]
Number of blood glucose concentrations <2.6 mmol/l per epoch	1 [1; 1]	1 [1; 2]	1 [1; 2]	1 [1; 2]	2 [1; 2]
Hours to maximum IG	3.5 (1.8) 3.0 [2.1; 5.3]	3.4 (1.6) 3.1 [2.2; 4.8]	3.0 (1.4) 2.7 [2.0; 4.1]	3.7 (1.8) 4.1 [2.2; 5.5]	3.5 (1.8) 3.5 [2.0; 5.3]
Average IG*	3.0 (0.3) 2.9 [2.8; 3.2]	3.1 (0.5) 3.0 [2.7; 3.3]	3.2 (0.5) 3.2 [2.8; 3.6]	3.5 (0.5) 3.4 [3.0; 3.6]	3.4 (0.9) 3.2 [2.8; 3.6]
Maximum IG^***^	3.5 (0.4)^a^ 3.4 [3.2; 3.6]	3.8 (1.0)^a,b^ 3.6 [3.3; 4.2]	3.9 (0.7)^a,b^ 3.7 [3.4; 4.3]	4.2 (0.8)^b,c^ 4.0 [3.7; 4.5]	4.6 (1.6)^c^ 4.0 [3.5; 5.5]
Minimum IG*	2.3 (0.2)^a^ 2.3 [2.1; 2.4]	2.2 (0.3)^a,b^ 2.2 [1.9; 2.4]	2.2 (0.3)^a^ 2.2 [2.1; 2.5]	2.2 (0.3)^a,b^ 2.2 [2.1; 2.4]	2.0 (0.4)^b^ 2.2 [1.9; 2.3]
IG range^***^	1.2 (0.5)^a^ 1.1 [0.8; 1.4]	1.6 (1.0)^a,b^ 1.3 [1.1; 1.9]	1.6 (0.7)^a,b^ 1.5 [1.1; 1.9]	2.0 (0.9)^b,c^ 1.8 [1.5; 2.4]	2.6 (1.6)^c^ 1.9 [1.4; 3.3]
Proportion IG outside central band of 3–4 mmol/l	0.54 (0.31) 0.57 [0.22; 0.81]	0.59 (0.26) 0.60 [0.40; 0.77]	0.51 (0.27) 0.55 [0.27; 0.72]	0.51 (0.28) 0.49 [0.27; 0.76]	0.68 (0.28) 0.75 [0.51; 0.92]
Duration (hours) IG <2.6 mmol/l*	1.00 (0.81) 0.64 [0.41; 1.30]	1.37 (1.12) 1.14 [0.49; 1.92]	1.17 (1.13) 0.72 [0.36; 1.50]	1.24 (1.15) 0.87 [0.41; 1.38]	1.91 (1.54) 1.43 [0.75; 2.80]

Data are number, median [interquartile range] or mean (standard deviation). ^†^Analysis restriction to babies born at ≥ 35 weeks’ gestation. Glucose concentrations are expressed as mmol/l. IG, interstitial glucose concentration. *P < 0.05, **P < 0.01, ***P < 0.001 for comparisons among treatments; letters indicate pairwise comparisons (Tukey adjustment).

Blood glucose concentration at onset was significantly lower in epochs in which IV dextrose was administered than in epochs treated with breast milk alone (MD −0.22 [95% CI −0.41; −0.02], P = 0.02, Table 5). Epochs in which IV dextrose was administered had higher IG maximum compared with those treated with breast milk alone (MD 1.18 mmol/l [95% CI 0.70; 1.66], P < 0.0001), breast milk plus dextrose gel (MD 0.78 mmol/l [95% CI 0.34; 1.21], P = 0.004) and formula alone (MD 0.79 mmol/l [95% CI 0.34; 1.24], P = 0.006, Table 5). Similarly, epochs treated with formula plus dextrose gel had higher IG maximum than those treated with breast milk alone (MD 0.71 mmol/l [95% CI 0.24; 1.18], P = 0.03; Table 5).

Epochs in which IV dextrose was administered also had lower IG minimum compared to those treated with breast milk alone (MD −0.23 mmol/l [95% CI −0.37; −0.07], P = 0.02) or breast milk plus dextrose gel (MD −0.20 mmol/l [95% CI −0.34; −0.07], P = 0.03, Table 5).

Epochs in which IV dextrose was administered had a higher IG range compared to those treated with breast milk alone (MD 1.40 mmol/l [95% CI 0.91; 1.89], P < 0.0001), with breast milk plus dextrose gel (MD 0.98 mmol/l [95% CI 0.54; 1.43], P = 0.0002) or with formula alone (MD 0.94 mmol/l [95% CI 0.48; 1.40], P = 0.0007; Table 5). Similarly, epochs treated with formula plus dextrose gel had a higher IG range than breast milk alone (MD 0.79 mmol/l [95% CI 0.31; 1.28], P = 0.01).

### Dextrose gel dose

Babies born ≥35 weeks’ gestational age who received >200 mg/kg of dextrose gel (≥2 doses) per epoch were not different from babies who received ≤200 mg/kg (1 dose) in socio-demographic and neonatal variables (data not shown). Blood glucose concentration at onset was significantly lower in epochs in which two or more dextrose gel doses were administered than epochs treated with one dextrose gel dose (Table 6). Administration of two or more dextrose gel doses compared with one dose was also associated with lower epoch IG average and minimum, higher proportion of time spent outside central 3–4 mmol/l glucose band and more time with IG < 2.6 mmol/l (Table 6).

**Table 6:** Effect of dextrose gel dose on interstitial glucose parameters in 6-hour epochs following hypoglycemia.

	Dextrose gel dose		
	≥2 doses (>200 mg/kg)	1 dose (≤200 mg/kg)	Mean difference [95% Confidence Intervals]
Number of 6-hour epochs	32	65	
Number of babies	22	46	
Blood glucose at the onset of the epoch*	2.1 (0.3) 2.2 [1.9; 2.3]	2.2 (0.3) 2.3 [2.1; 2.5]	−0.15 [−0.28; −0.01]
Number of blood glucose concentration <2.6 mmol/l per epoch	2 [2; 3]	1 [1; 1]	
Hours to maximum IG	3.8 (1.7) 3.7 [2.4; 5.4]	3.2 (1.6) 3.0 [2.0; 4.4]	0.55 [−0.15; 1.24]
Average IG**	3.0 (0.5) 3.0 [2.6; 3.3]	3.4 (0.6) 3.3 [3.0; 3.7]	−0.39 [−0.62; −0.15]
Maximum IG	4.1 (1.3) 3.8 [3.4; 4.3]	4.1 (0.9) 4.0 [3.4; 4.6]	−0.04 [−0.50; 0.41]
Minimum IG*	2.1 (0.4) 2.2 [1.8; 2.3]	2.2 (0.3) 2.3 [2.1; 2.5]	−0.17 [−0.32; −0.02]
IG range	2.0 (1.5) 1.6 [1.2; 2.1]	1.9 (0.9) 1.6 [1.3; 2.4]	0.12 [−0.36; 0.61]
Proportion IG outside central band of 3–4 mmol/l*	0.63 (0.30) 0.68 [0.37; 0.90]	0.51 (0.27) 0.53 [0.28; 0.72]	0.12 [0.00; 0.24]
Duration (hours) IG <2.6 mmol/l^***^	2.28 (1.48) 1.91 [1.18; 3.63]	0.97 (0.97) 0.65 [0.39; 1.28]	0.87 [0.51; 1.23]

Data are number, median [interquartile range], mean (standard deviation), or mean difference [95% confidence intervals]. IG, interstitial glucose concentration. Glucose concentrations are expressed as mmol/l. *P < 0.05, **P < 0.01, ^***^P < 0.001 for comparison between ≥ 2 and 1 gel doses.

### IV dextrose

Of 46 epochs where IV dextrose was administered, a bolus as well as a continuous infusion was administered in 29 (63%), a bolus was administered without ongoing infusion in three (7%), and dextrose gel was administered in 19 (41%). Of 43 epochs where only continuous dextrose infusion was administered, in 19 (44%), the infusion had been started prior to the onset of the epoch. In the remaining 24 (56%), the IV dextrose was administered at a median of 0.70 [0.37; 1.00] hours from the onset of the epoch.

Among babies born at 32–34 weeks’ gestational age receiving IV dextrose, there was no relationship between epoch IG parameters and GDR (mg/kg/min), use of IV bolus or dextrose gel (Table 7). In babies born at ≥35 weeks’ gestational age treated with IV dextrose, there was also no relationship between epoch IG parameters and GDR. Use of IV bolus was associated with lower epoch IG minimum (MD −0.33 mmol/l [95% CI −0.60; −0.07], P < 0.05) but not any other epoch IG parameters. Dextrose gel administration was associated with lower epoch IG average (MD −0.76 mmol/l [95% CI −1.46; −0.06], P < 0.05) and IG minimum (MD −0.39 mmol/l [95% CI −0.69; −0.08], P < 0.05), and increased proportion of time outside central band of 3–4 mmol/l (MD 0.25 [95% CI 0.02; 0.48], P < 0.05) and time <2.6 mmol/l (MD 1.03 hours [95% CI 0.34; 1.72], P < 0.01) (Table 7).

**Table 7:** Interstitial glucose parameters in 6-hour epochs with intravenous dextrose treatment.

	Bolus (yes vs no)	GDR (mg/kg/min)	Dextrose gel (yes vs no)
**32**–**34 weeks’ gestational age:**			
Epochs (babies) N = 10 (9)			
Hours to maximum IG	0.44 [−1.70; 2.58]	−0.12 [−0.44; 0.19]	−1.25 [−3.85; 1.34]
Average IG	−0.08 [−2.98; 2.82]	−0.09 [−0.53; 0.35]	0.33 [−3.10; 3.76]
Maximum IG	0.05 [−5.68; 5.78]	0.00 [−0.83; 0.84]	0.15 [−6.80; 7.12]
Minimum IG	−0.50 [−2.28; 1.28]	−0.17 [−0.50; 0.10]	−0.12 [−2.21; 1.96]
IG range	0.85 [−4.70; 6.39]	0.11 [−0.70; 0.91]	0.45 [−6.27; 7.17]
Proportion IG outside central band of 3–4 mmol/l	0.30 [−0.36; 0.97]	0.07 [−0.03; 0.17]	0.67 [−0.12; 1.46]
Duration (hours) IG <2.6 mmol/l	1.46 [−0.52; 3.44]	0.21 [−0.10; 0.51]	0.02 [−1.49; 3.14]
**≥35 weeks’ gestational age:**			
Epochs (babies) N = 36 (19)			
Hours to maximum IG	−0.53 [−1.87; 0.82]	−0.08 [−0.35; 0.20]	−0.16 [−1.71; 1.39]
Average IG	−0.23 [−0.83; 0.36]	0.00 [−0.12; 0.13]	−0.76 [−1.46; −0.06]*
Maximum IG	0.04 [−1.12; 1.19]	−0.05 [−0.29; 0.19]	−0.38 [−1.74; 0.97]
Minimum IG	−0.33 [−0.60; −0.07]*	0.00 [−0.05; 0.06]	−0.39 [−0.69; −0.08]*
IG range	0.37 [−0.82; 1.56]	−0.06 [−0.31; 0.19]	0.00 [−1.39; 1.39]
Proportion IG outside central band of 3–4 mmol/l	0.09 [−0.11; 0.28]	0.02 [−0.02; 0.06]	0.25 [0.02; 0.48]*
Duration (hours) IG < 2.6 mmol/l	0.39 [−0.21; 0.99]	−0.02 [−0.14; 0.10]	1.03 [0.34; 1.72]**

Data are regression coefficient [95% confidence interval]. Adjusted for time when IV was started; IG, interstitial glucose concentration; GDR, glucose delivery rate. Glucose concentrations are expressed as mmol/l. *P < 0.05, **P < 0.01.

### Neurosensory impairment

**Figure 1:**
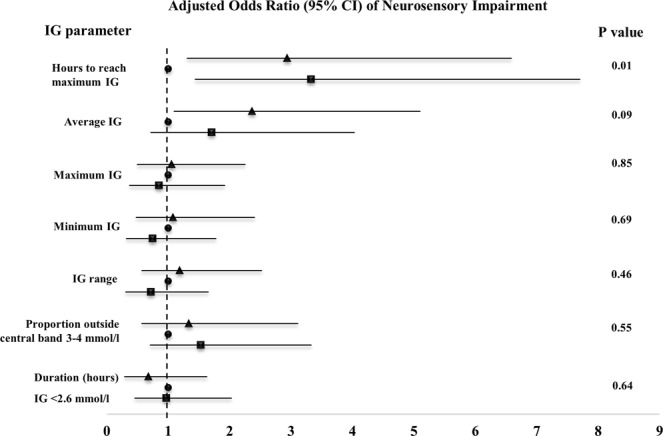
Association between interstitial glucose (IG) parameters in six-hour epochs following neonatal hypoglycaemia and neurosensory impairment in early childhood. Exposure effect is presented as odds of neurosensory impairment by tertile of IG parameter (referent middle tertile). Results adjusted for socio-economic status, gestational age, birth weight Z score and blood glucose concentration at the beginning of the epoch. Triangles denote tertile 1, circles tertile 2 (reference) and squares tertile 3. Tertile values are as follows: hours to reach maximum IG (1: 0.4–2.2; 2: 2.3–4.2; 3: 4.3–6.0), average IG (1: 1.7–2.9; 2: 2.9–3.3; 3: 3.4–6.2 mmol/L), maximum IG (1: 2.6–3.4; 2: 3.5–4.0; 3: 4.1–8.7 mmol/L), minimum IG (1: 0.1–2.1; 2: 2.1–2.3; 3: 2.4–2.8 mmol/L), IG range (1: 0.3–1.2; 2: 1.3–1.9; 3: 2.0–7.0 mmol/L), proportion of IG measurements outside central band of 3–4 mmol/L (1: 0.06–0.39; 2: 0.40–0.68; 3: 0.69–1.00), hours <2.6 mmol/L (1:0.2–0.5; 2: 0.6–1.3; 3: 1.4–5.9).

The risk of neurosensory impairment was increased with both shorter and longer time (hours) to reach maximum epoch IG (P = 0.04; post hoc Dunnett adjusted pairwise comparison lower tertile [0.4–2.2 hours] vs middle [2.3–4.2 hours] OR 3.10 [95% CI 1.03; 9.38], P = 0.04; higher tertile [4.3–6.0 hours] vs middle OR 3.07; [95% CI 1.01; 9.24], P = 0.04) (Fig. 1). Results were similar without adjustment for socio-economic status, gestational age, birth weight Z score and blood glucose concentration at onset of epoch (P = 0.03). Neurosensory impairment was not associated with other IG parameters. In a sensitivity analysis, exclusion of babies born at 32–34 weeks’ gestational age did not alter results.

Because of a potentially complex relationship between the degree of hypoglycaemia, glycaemic instability and neurosensory outcome, *post hoc* interaction tests were performed to determine if epoch IG minimum influenced the association between neurosensory impairment and time to reach maximum epoch IG and proportion of IG concentrations outside the central band of 3 to 4 mmol/l, but these tests were not significant (P = 0.3 and P = 0.2, respectively).

Further *post hoc* analysis was undertaken to determine if tertiles of time to reach maximum epoch IG were related to the severity of hypoglycaemia, glycaemic instability and treatment of the epoch. Tertiles for time to reach maximum epoch IG did not differ significantly by epoch IG minimum (tertile 1, 2, 3 mean [SD][range]: 2.2 [0.5][0.1, 2.6], 2.2 [0.4][0.6, 2.8], 2.2 [0.3][1.4, 2.7] mmol/l; P = 0.99), IG maximum (tertile 1, 2, 3 mean [SD][range]: 3.9 [0.8][2.8, 6.5], 4.2 [1.1][2.6, 8.7], 3.8 [0.9][2.7, 8.7] mmol/l; P = 0.16), IG range (tertile 1, 2, 3 mean [SD][range]: 1.7 [1.1][0.7, 5.6], 2.1 [1.3][0.4, 7.9], 1.7 [1.1][0.3, 6.4]; P = 0.19), and proportion of IG concentrations outside the central band of 3 to 4 mmol/l (tertile 1, 2, 3 mean [SD][range]: 0.57 [0.29][0.06, 1.00], 0.53 [0.28][0.08, 1.00], 0.54 [0.28][0.07, 1.00] mmol/l; P = 0.76). The proportion of babies with an intermediate rate of correction of hypoglycaemia (middle tertile for time to reach maximum epoch IG) did not differ significantly by treatment group but was highest in those treated with breast milk and buccal dextrose gel (breast milk only 24%, formula 38%, breast milk plus buccal dextrose gel 47%, formula plus buccal dextrose gel 23%, and IV dextrose 21%; P = 0.29).

## Discussion

In this study we found that parameters reflecting glucose stability in 6-hour epochs following hypoglycaemia in the first 48 hours were not related to neonatal risk factors but were related to treatment. However, only the rate of change in glucose concentrations was related to adverse neurodevelopmental outcome.

Results of the CHYLD study showed that higher glucose concentrations, even within the normal range, and also less stable glucose concentrations, indicated by increasing proportion of measurements outside the central band of 3 to 4 mmol/l (54 to 72 mg/dl) were associated with higher risk of neurosensory impairment at two years^8^. This finding is consistent with animal studies showing that brain injury is more severe when low blood glucose concentrations are followed by hyperglycaemia^20^. However, in our previous study, while babies who experienced hypoglycaemia had a greater proportion of blood glucose concentrations outside the central band, the extent to which instability was temporally related to hypoglycaemia and also to the subsequent higher glucose concentrations after hypoglycaemia was unclear^8^.

Our detailed analysis found that those with the most unstable blood glucose concentrations in the first 48 hours also had less stable glucose concentrations in the period immediately after hypoglycaemia. Further, this instability was due not only to low but also to high glucose concentrations. Although we did not find an association between the proportion of epoch IG measurements outside the central band and neurosensory impairment, we did find that glycaemic responses immediately following hypoglycaemia were associated with overall stability of blood glucose concentrations. This suggests that to achieve greater stability of neonatal blood glucose concentrations, attention needs to be directed to the period after the onset of hypoglycaemia.

Variation in glucose stability among babies at risk of hypoglycaemia may reflect differences in underlying pathophysiology of hypoglycaemia in different risk groups. For example, babies born to diabetic mothers are thought to have an increased transfer of fuels across placenta due to maternal hyperglycaemia, resulting in hyperinsulinism^21,22^. Another mechanism is described in preterm babies, who might not have sufficient intrauterine time to deposit fat and glycogen or have accompanying conditions that affect glucose production^5^. However, we found no association between primary neonatal risk factor and epoch IG parameters. Thus, among at-risk infants, glycaemic response to hypoglycaemia does not appear to be related to underlying risk.

Nevertheless, the most immature babies born at 32–34 weeks’ gestational age had the least stable epoch IG parameters (average, maximum, minimum and range) even though most received IV dextrose which might have been expected to stabilise IG concentrations. Preterm babies have been previously reported to have poor adaptation mechanisms to postnatal life and therefore have low blood glucose concentrations after birth^2^. Immaturity of metabolic systems makes the management of glucose concentrations in these babies difficult, and treatment of hypoglycaemia often results in hyperglycaemia^23^. Very preterm babies were found to have unstable IG concentrations even after they were clinically stable and receiving full enteral feeds, with the instability more pronounced in less mature babies^24^. Therefore, babies born <35 weeks’ gestational age seem to be at high risk for glycaemic instability and require close monitoring.

We wanted to establish if the instability was related to different responses of babies who were treated similarly or to different treatments. We found no differences in epoch IG parameters treated with breast milk alone or formula alone, in contrast to a previous report that babies born to mothers with gestational diabetes had higher blood glucose concentrations after receiving formula than after breast feeding^25^. Moreover, we found that treatment with dextrose gel plus breast milk was not associated with glucose instability, whereas treatment with formula plus dextrose gel or IV dextrose was associated with instability.

Epochs in which IV dextrose was administered had lower blood glucose concentrations at the onset of the epoch, which might explain why babies received more aggressive treatment, possibly to increase glucose concentrations fast and achieve stabilisation. However, IG parameters for these epochs were more unstable than other treatment group epochs, and were characterised by both low and high glucose concentrations and more time outside central band and time below IG 2.6 mmol/l. Again, it is possible that babies who spent more time below 2.6 mmol/l received more aggressive treatment and in different forms. Another possible explanation might be individual variation in metabolic response to treatment of hypoglycaemia, whereby babies with blood glucose concentrations in the lowest range have less developed homeostatic systems that are not able to maintain stable glucose concentrations when dextrose is administered rapidly.

Among epochs where dextrose gel was administered, two or more dextrose gel doses were associated with lower blood glucose concentration at the start of the epoch, lower average, minimum and more time spent outside the central band and below IG 2.6 mmol/l, but similar maximum IG. This suggests that instability in these epochs was due to low glucose concentrations and treatment with multiple doses of dextrose gel did not result in high glucose concentrations. Like IV dextrose treatment, a more aggressive treatment might have been chosen for babies whose glucose concentration was low at the start of the epoch and remained low.

Literature on the associations between feeding, dextrose treatment and glucose concentrations is limited, and factors that might identify babies who have different metabolic responses to different treatments are unclear. In very preterm babies studied at term equivalent age, IG concentrations fluctuated (both hypo- and hyperglycaemic), with no differences between breast fed and formula fed babies, and no association with specific risk factors^26^. Moreover, blood glucose concentrations were not different between breast and formula fed small-for-gestational-age babies, but the concentration of ketone bodies was higher in breast fed babies^21^. Further, blood glucose response to mini-bolus followed by intravenous infusion was different in babies who were average or small for gestational age, or large/infants of diabetics, but variation within each group was also described^27^. In addition, treatment of hypoglycaemia with dextrose gel or formula led to increase in glucose concentrations, while breast feeding did not^28^. However, breast feeding was associated with decreased requirement for a second treatment of hypoglycaemia.

Similar to these reports, our data suggests that IG parameters and treatment of hypoglycaemia are related, but we cannot establish the sequence of events to determine causality. This study does not allow us to predict which babies are most at risk of glycaemic instability or predict their response to treatment. However, we can suggest that if oral treatment of hypoglycaemia is planned, preference might be given to breast milk or breast milk plus dextrose gel over formula plus dextrose gel, which was associated with greater instability, as well as lacking the other benefits of breast feeding^29^.

Our previous report of the increased rate of neurosensory impairment in babies with higher and less stable glucose concentrations is consistent with other reports that glucose instability was associated with adverse health outcomes and increased mortality rates in very low birth weight babies^30^ and adults^31,32^. Although in this subgroup analysis, glucose stability, as represented by the proportion of epoch IG within the central band, was not related to outcome, we found an association between the rate of change in IG after hypoglycaemia (time to IG maximum) and neurosensory impairment, which was independent of the severity of hypoglycaemia. Further, this relationship appeared U-shaped which suggests that close monitoring is needed at the time of treatment to ensure the appropriate rate of change in glucose concentrations. While time to IG maximum was not itself associated with neonatal risk factors or different types of feeding and treatment, babies treated with breast milk and dextrose gel appeared to be more likely to have an intermediate rate of correction of hypoglycaemia than other forms of treatment.

An important strength of our study is the use of interstitial glucose monitoring that records glucose concentrations continuously and provides readings every five minutes, but these were not used for the management of neonatal hypoglycaemia. Therefore, we could analyse associations between treatment and IG parameters in epochs of babies treated according to clinical guidelines and not influenced by our findings.

One limitation of CGM is the delay in obtaining data after birth, resulting from the practical difficulties of inserting the monitor immediately after birth, and the delay between insertion of the glucose monitor and obtaining the first readings. This means that few data are available in the first critical one to three hours after birth, which is a time when blood glucose concentrations are most commonly low^33^. Further, while this is the largest cohort of term and late preterm babies born at-risk of hypoglycaemia with GCM, we had limited power to assess interactions between degree of hypoglycaemia, glycaemic instability, rate of correction and neurosensory outcome. These relationships may be complex and larger samples or randomised intervention trials will be required to fully elucidate pathways from neonatal dysglycaemia to abnormal neurosensory development.

In this study we have demonstrated that babies who have unstable blood glucose concentrations in the 48 hours after birth also have unstable glucose concentrations in the period immediately after the onset of hypoglycaemia. Glycaemic responses after hypoglycaemia were not related to neonatal risk factors but were related to treatment. Intravenous dextrose administration was associated with both low and high glucose concentrations after hypoglycaemia, whereas multiple dextrose gel doses were associated with low but not high concentrations. Rate of change in glucose concentrations, both fast and slow, appears to be associated with neurosensory impairment. These findings suggest that interventions that help stabilise glucose parameters in the period after the onset of hypoglycaemia may be associated with improved neurosensory outcomes after neonatal hypoglycaemia.

## Data Availability

Published data are available to approved researchers under the data sharing arrangements provided by the Maternal and Perinatal Central Coordinating Research Hub (CCRH), based at the Liggins Institute, University of Auckland (https://wiki.auckland.ac.nz/researchhub). Metadata, along with instructions for data access, are available at the University of Auckland’s research data repository, Figshare (https://auckland.figshare.com). Data access requests are to be submitted to Data Access Committee via researchhub@auckland.ac.nz.
